# Supplementary material for: NXC736 Attenuates Radiation-Induced Lung Fibrosis via Regulating NLRP3/IL-1β Signaling Pathway
Source: Int J Mol Sci. 2023 Nov 13;24(22):16265. doi: 10.3390/ijms242216265 (PMC10671169; doi:10.3390/ijms242216265)
Supplement: Supplementary file 1 [file ijms-24-16265-s001.zip › ijms-2616039-supplementary.pdf]

Supplementary data

Supplementary Figure S1. The change of mice body weight. There was no significant difference between the groups. Data are expressed as the mean ± standard deviation.

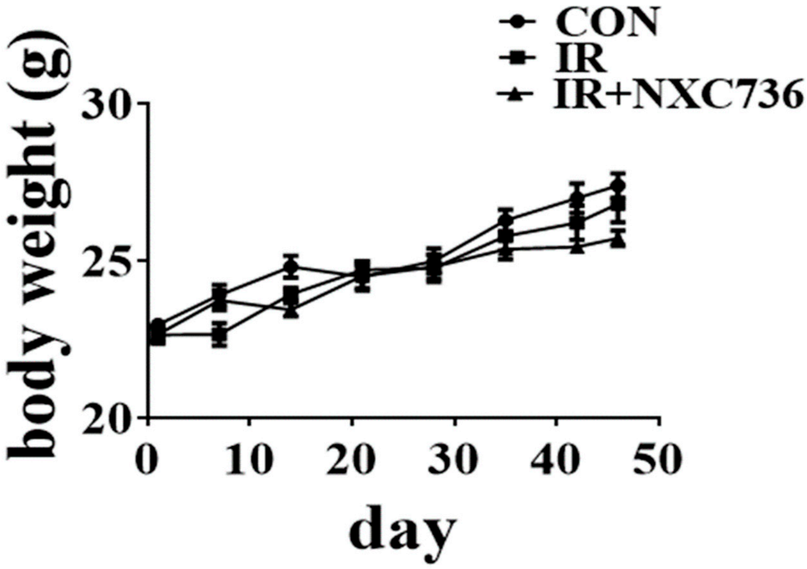

Supplementary Figure S2. The change in expression levels of cleaved caspase-1 and caspase-1. The cleaved caspase-1 was detected using the cleaved caspase-1 antibody purchased from Gentex in left pannel. The Caspase-1 was detected using caspase-1 antibody purchased from Abcam in right pannel. GAPDH was also detected to loading normalization.

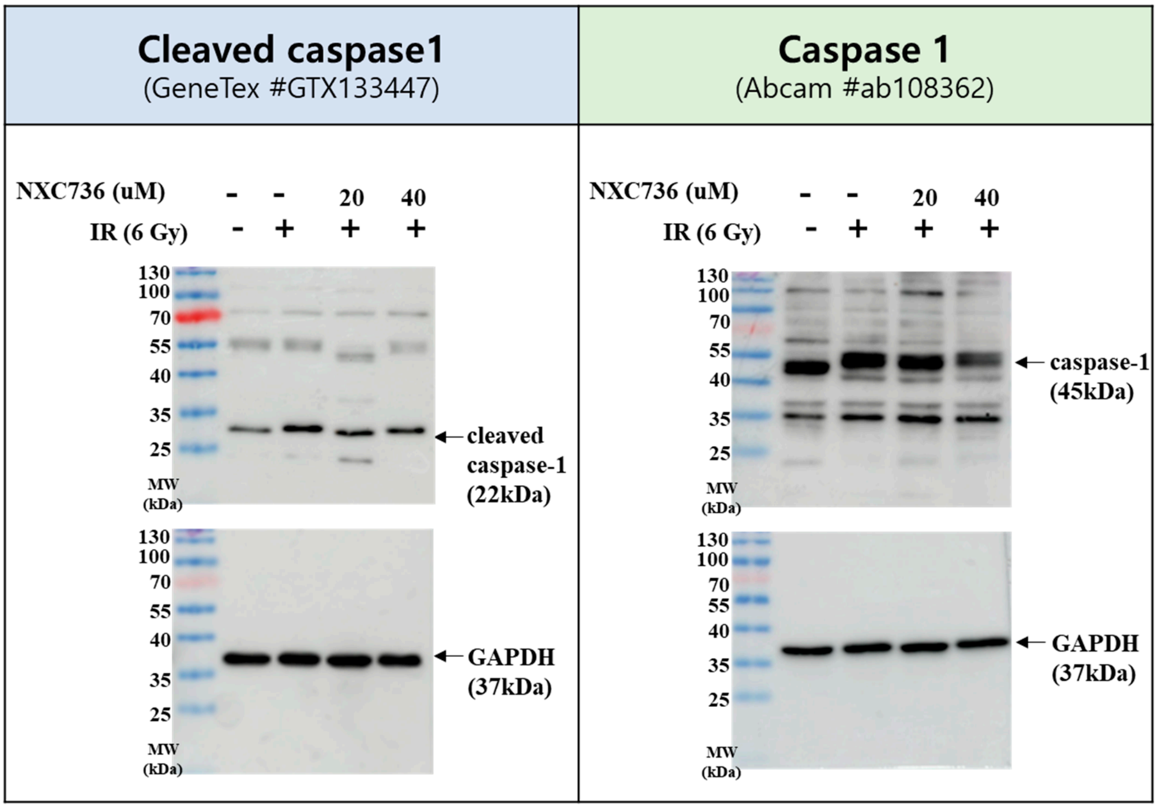

Supplementary Figure S3. L132 cells were irradiated with a dose of 6 Gy, harvested 24 hours later, and the expression of S1PR1 and S1PR4 at the mRNA level was measured using the q-PCR method. NXC736 altered the expression of S1PR1 and S1PR4 at the mRNA level. Data are expressed as the mean ± standard deviation (**p* < 0.05, ****p* < 0.001, and *****p* < 0.0001).

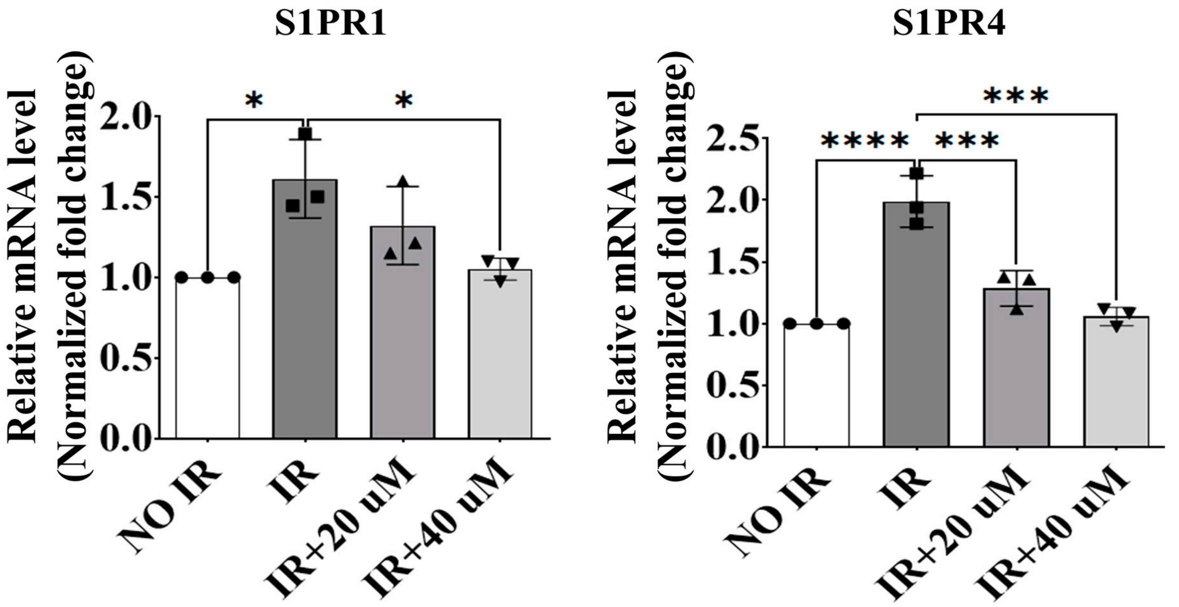

Supplementary Figure S4. Uncut Western blot image in Figure 3. Yellow box is original image in figure 3(C) and (D). The experiment was repeated 3 times (n = 3; remarked #1, #2, #3). #1 is a representative image of this paper. The red-dashed box is the target antibody band being detected, and the size of the target antibody band can be confirmed using the size marker.

Figure 3.	Ab list	#1	#2	#3
Figure 3(C) 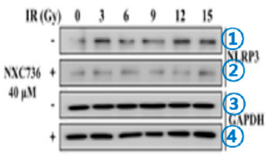	① anti-NLRP3 (118kDa)	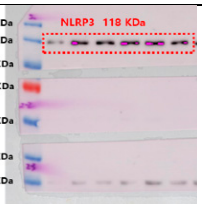	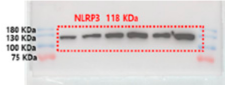	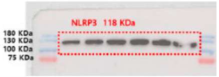
	② anti-NLRP3 (118kDa)	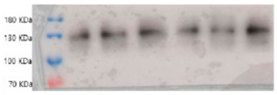	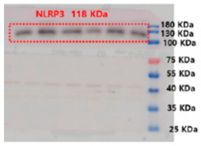	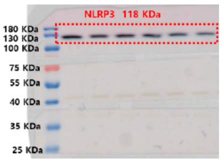
	③ anti-GAPDH (37kDa)	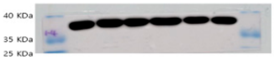	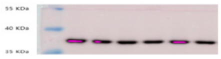	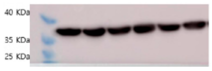
	④ anti-APDH (37kDa)	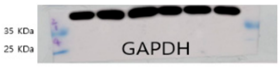	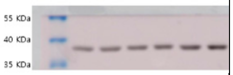	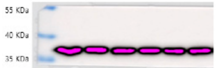
Figure 3(D) 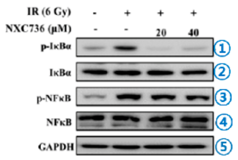	① anti-IKBα (36 kDa)	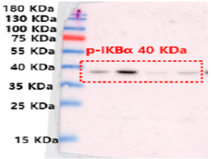	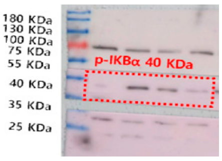	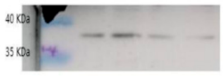
	② anti-IKBα (36 kDa)	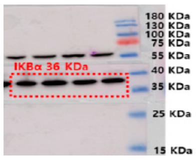	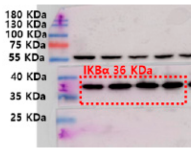	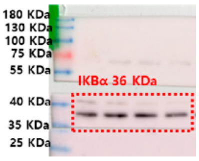
	③ anti-p-NFkB (65kDa)	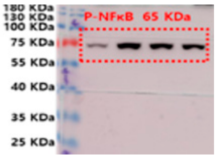	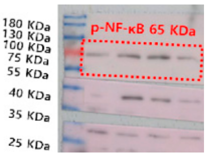	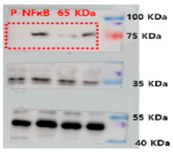
	④ anti-NFkB (65kDa)	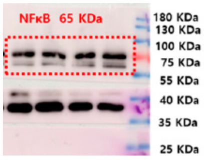	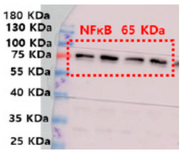	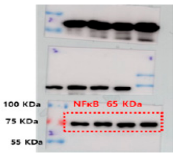
	⑤ anti-GAPDH (37kDa)	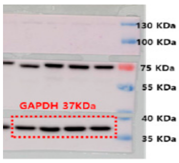	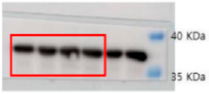	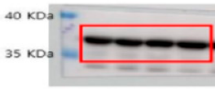

Supplementary Figure S5. Western blot uncut image in Figure 4. Yellow box is original image in figure 4(D). The experiment was repeated 3 times (n = 3; remarked #1, #2, #3). #1 is a representative image of this paper. The red-dashed box is the target antibody band being detected, and the size of the target antibody band can be confirmed using the size marker.

Figure 4(D)	Ab list	#1	#2	#3
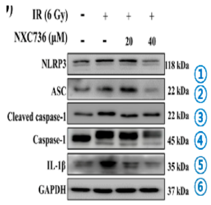	① Anti-NLRP3 (118 kDa)	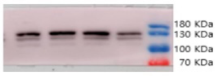	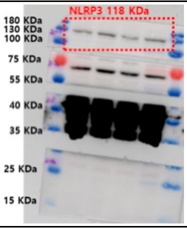	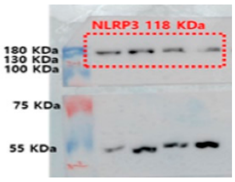
	② Anti-ASC (22 kDa)	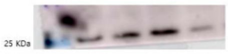	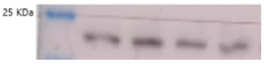	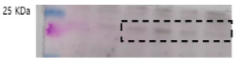
	③ anti-Cleaved caspase-1 (22 kDa)	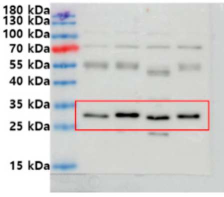	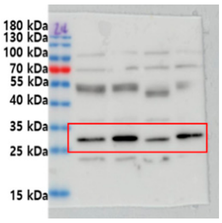	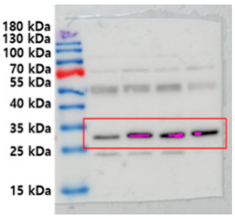
	④ anti-Caspase-1 (45 kDa)	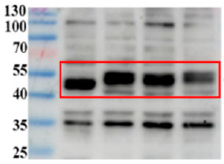	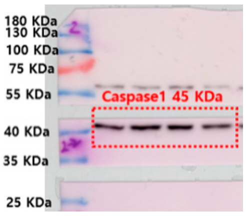	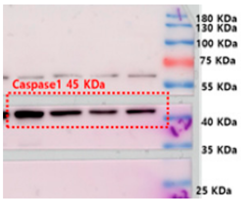
	⑤ anti-IL-1β (35 kDa)	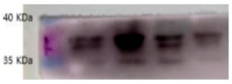	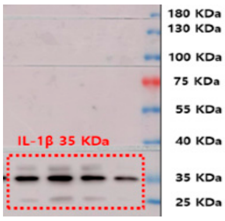	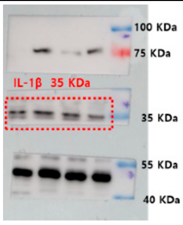
	⑥ anti-GAPDH (37 kDa)	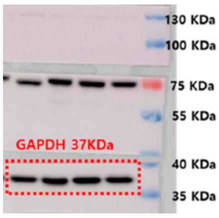	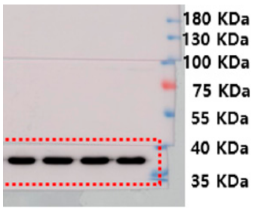	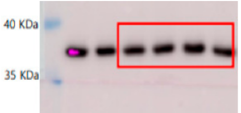

Supplementary Figure S6. Western blot uncut image in Figure 5. Yellow box is original image in figure 5(B). The experiment was repeated 3 times (n = 3; remarked #1, #2, #3). #1 is a representative image of this paper. The red-dashed box is the target antibody band being detected, and the size of the target antibody band can be confirmed using the size marker.

<table>
<tr><th>Figure 5(B)</th><th>Ab list</th><th>#1</th><th>#2</th><th>#3</th></tr>
<tr><td rowspan="9">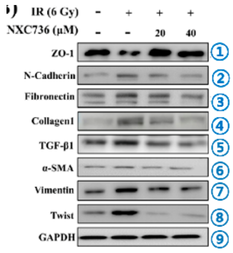
</td><td>① anti-ZO-1 (220 kDa)</td><td>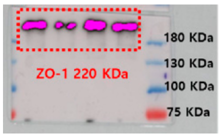
</td><td>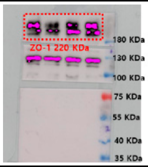</td><td>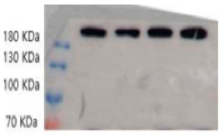</td></tr>
<tr><td>② anti-N-Cadherin (140 kDa)</td><td>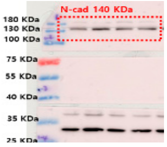</td><td>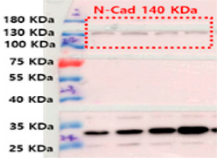
</td><td>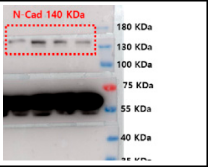</td></tr>
<tr><td>③ anti-Fibronectin (280 kDa)</td><td>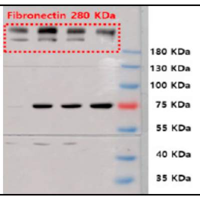
</td><td>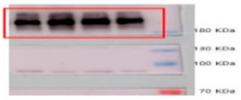</td><td>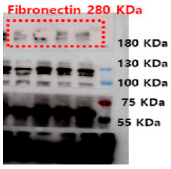
</td></tr>
<tr><td>④ anti-Collagen1 (138 kDa)</td><td>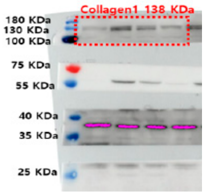
</td><td>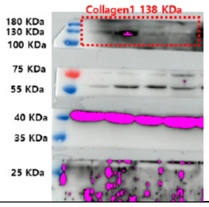
</td><td>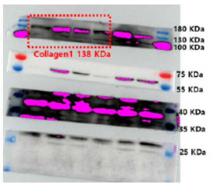</td></tr>
<tr><td>⑤ anti-TGF-β1 (44 kDa)</td><td>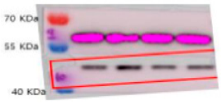</td><td>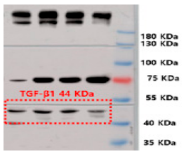
</td><td>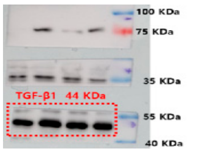
</td></tr>
<tr><td>⑥ anti-α-SMA (42 kDa)</td><td>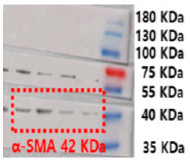
</td><td>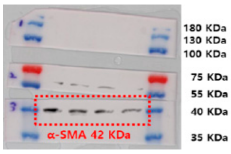
</td><td>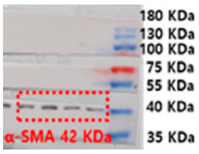
</td></tr>
<tr><td>⑦ anti-Vimentin (57 kDa)</td><td>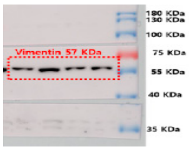</td><td>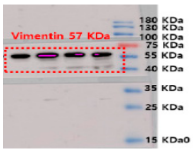
</td><td>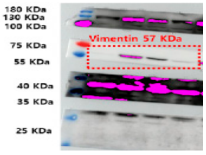
</td></tr>
<tr><td>⑧ anti-twist (22 kDa)</td><td>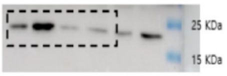
</td><td>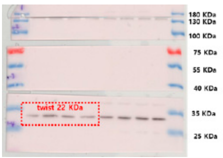</td><td>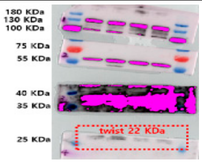
</td></tr>
<tr><td>⑨ anti-GAPDH (37 kDa)</td><td>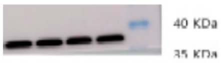
</td><td>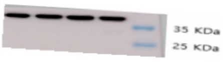
</td><td>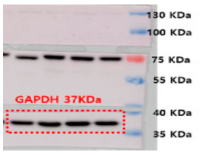
</td></tr>
</table>

Supplementary Figure S7. Western blot uncut image in Figure 6(B). Yellow box is original image in figure 6(B). The experiment was repeated 3 times (*n* = 3; remarked #1, #2, #3). #1 is a representative image of this paper. The red-dashed box is the target antibody band being detected, and the size of the target antibody band can be confirmed using the size marker.

Figure 6(B)	Ab list	#1	#2	#3
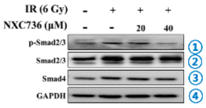	① anti-p-Smad2/3 (60 kDa)	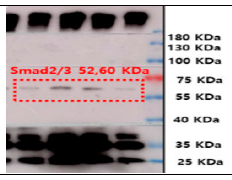	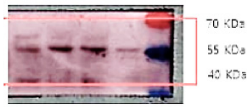	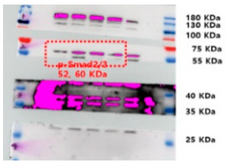
	② anti-Smad2/3 (60 kDa)	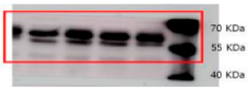	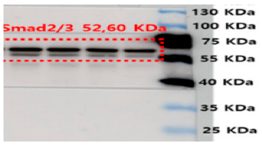	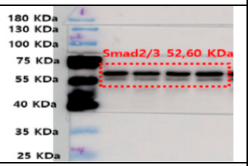
	③ anti-Smad4 (70 kDa)	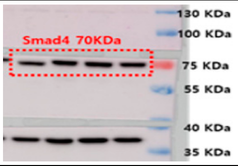	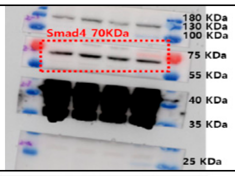	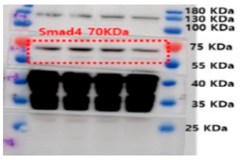
	④ anti-GAPDH (37 kDa)	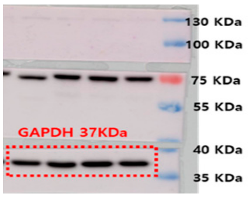	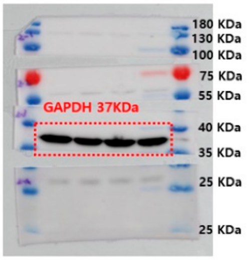	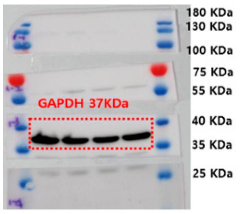

Supplementary Table S1. The parameter description of the flexiVent™ system measurements

Abbreviations	Parameter	Description
IC	Inspiratory Capacity	Amount of air that can be inhaled after the end of a normal expiration.
Rrs	Resistance of the respiratory system	Dynamic resistance quantitatively assesses the level of constriction in the lungs.
Crs	Compliance of the respiratory system	Compliance (also known as dynamic compliance) describes the ease with which the respiratory system can be extended. In a subject with intact chest walls, it provides a characterisation of the overall elastic properties that the respiratory system needs to overcome during tidal breathing to move air in and out of the lungs.
Ers	Elastance of the respiratory system	Elastance captures the elastic stiffness of the respiratory system at the ventilation frequency. If measured under closed-chest conditions, it includes a contribution from the lung, the chest walls, and the airways. Elastance is the reciprocal of compliance and vice versa.
Rn	Newtonian Resistance	Parameter of the Constant Phase Model which represents the resistance of the central or conducting airways.
G	Tissue Damping	Parameter of the Constant Phase Model closely related to tissue resistance and reflects the energy dissipation in the alveoli.
H	Tissue Elastance	Parameter of the Constant Phase Model closely related to tissue elastance and reflects the energy conservation in the alveoli.
Cst	Quasi-static Compliance	Quasi-static compliance is a classic parameter extracted from a PV curve. If measured under closed-chest conditions, it reflects the intrinsic elastic properties of the respiratory system (i.e. lung+chest wall) at rest.

Supplementary Table S2. Primer sequences used for real-time polymerase chain reaction.

Gene	Forward (5’ →3’)	Reverse (5’ →3’)
TNF-α	GGAGAAGGGTGACCGACT	CTGCCCAGACTCGGCAA
IL-6	GTCAGGGGTGGTTATTGC	AGTGAGGAACAAGCCAGA
IL-18	CAGCCTAGAGGTATGGCTGT	TCATGTCCTGGGACACTTCTC
TGF-β	GCAGCACGTGGAGCTGTA	CAGCCGGTTGCTGAGGTA
NLRP3	CACCTGTTGTGCAATCTGAAG	GCAAGATCCTGACAACATGC
IL-1β	TACCTGTCCTGCGTGTTGAAA	CTGCTTGAGAGGTGCTGATGT
S1PR1	TATCAGCGCGGACAAGGAGAACAG	ATAGGCAGGCCACCCAGGATGAG
S1PR4	GACGCTGGGTCTACTATTGCC	CCTCCCGTAGGAACCACTG

Cycling conditions: Denature: 95°C for 30 s, 95°C for 4 min, followed by 35 cycles of 95°C for 10 s, 55–61°C (shown in column of Annealing Temperature) for 15 s and 72°C for 15 s
